# Stem cell activity shapes the pleiotropic effects of IFN-γ and TGF-β in autoimmune diseases, infections, and cancer, and drives autoimmune flares and remissions

**DOI:** 10.3389/fimmu.2025.1704642

**Published:** 2025-11-26

**Authors:** Zeev Elkoshi

**Affiliations:** Retired, Kfar Saba, Israel

**Keywords:** autoimmunity, relapse, remission, stem cells, IFN-γ, TGF-β, pleiotropic effect

## Abstract

This work introduces stem cell activity as a central factor contributing to the pleiotropic effects of IFN-γ and TGF-β1, as well as to the fluctuations of autoimmune diseases (AIDs) between flares and remissions. Analysis of published data on hair follicle immune privilege indicates that immune protection is not an inherent feature of quiescent stem cells, as previously proposed, but instead depends on the specific pathways that regulate quiescence. While both IFN-γ and high levels of TGF-β1 induce stem cell quiescence, they exert opposite effects on immune privilege: IFN-γ upregulates MHC-I expression, whereas TGF-β1 downregulates it. Similar mechanisms apply to hematopoietic stem cell niches in the bone marrow. Moreover, cytokines such as IGF-1 and α-MSH, which enhance stem cell activity, also downregulate MHC-I. Different concentrations and combinations of these cytokines can promote or suppress stem cell activity and preserve or disrupt immune privilege, underscoring their multifaceted nature. Two mechanisms may contribute to the pleiotropic effects of IFN-γ and TGF-β1: opposing effects on bone marrow activity, with IFN-γ and high TGF-β1 acting in contrast, and differential effects of IFN-γ on immune attack intensity in the bone marrow versus the target tissue during AID. Stem cell dynamics also shape the course of AIDs: high stem cell activity supports tissue regeneration and remission, whereas quiescence together with tissue destruction by autoimmune attacks drives flares. A clear correlation emerges between the effects of various agents on stem cell activity and clinical outcomes in AIDs, highlighting the central role of stem cell activity in their pathogenesis. A proposed TGF-β1 gradient between protected stem cell reservoirs (hair follicle bulge, bone endosteal niches) and less protected regions enables simultaneous preservation of stem cells and regeneration of damaged tissue.

## Introduction

An earlier publication recognized the balance between tissue regeneration and destruction rates during autoimmune diseases (AIDs) as a key factor influencing the fluctuation of symptoms between flare-ups and remissions (1). Equation 1 presents this balance as:

(1) R = Rreg −Rdes

Where *R* is the tissue net generation rate, *Rreg* is the regeneration rate of the tissue under the autoimmune attack, and *Rdes* is the destruction rate of the tissue due to the autoimmune attack.

The generation of tissue mass, however, is defined by the integral:

(2) ΔM=∫R(t)dt

Where: *M* is the tissue mass, *ΔM* is the change in tissue mass, *t* is the time, and *R*(*t*) is the time-dependent tissue generation rate.

When *R* > 0, autoimmune response should not evoke symptoms since tissue regeneration rate exceeds tissue destruction rate. Autoimmune symptoms appear when *R* < 0, and when this condition is sustained over time. This happens when the relation *Rreg* < *Rdes* is sustained. Under the last condition, all tissue cells will eventually be destroyed, if the immune attack continues. Under this condition the affected tissue totally loses its function over time, and no spontaneous remission (or relapse) is expected (since even if a new active tissue cell is generated, the continuous immune attack will destroy it). If the *R* value fluctuates frequently between positive and negative, the resulting *ΔM* in Equation 2 will approach zero over time.

The regeneration rate (*Rreg*) depends on the presence and activity of progenitor or stem cells, which supply new cells to replace those that have been destroyed. The destruction rate (*Rdes*) is determined by immune cell activity directed against the tissue—present as immune surveillance under normal conditions but intensified during infection, AIDS, or cancer. Tissues with high stem cell activity that come under autoimmune attack can recover, enabling patients to remain asymptomatic, sometimes for prolonged periods. Inflammatory bowel disease (IBD) is an example. The high renewal capacity of the intestines allows a large proportion of IBD patients to have mucosal inflammation without clinical symptoms (2). Indeed, intestinal stem cells can renew and differentiate into different intestinal cell types including absorptive enterocytes, mucus-secreting goblet cells, hormone-producing enteroendocrine cells, antimicrobial peptide-releasing Paneth cells, etc. (3). Alopecia areata (AA) is another example of an AID in which symptoms can disappear for extended periods. In AA, hair bulb stem cells are spared during the immune attack on hair follicle (HF) (4, 5). This property preserves the regenerative capacity of the hair follicle, allowing a remission which may follow by relapse. The anagen hair-bulb is considered an immune privilege (IP) organ, with a limited accessibility to the immune system (6). The collapse of immune privilege is considered as a key element in the pathogenesis of AA. Interferon gamma (IFN-γ) was reported as a driver of IP collapse whereas α-melanocyte stimulating hormone (α-MSH), insulin-like growth factor 1 (IGF-1), tacrolimus and transforming growth factor β1 (TGF-β1) have been shown to restore HF- IP (6–9). These effects were evaluated by examining the impact of these molecules on MHC-I expression in anagen HF epithelium (9), based on the assumption that CD8+ T cells are the primary effectors of the autoimmune attack in AA.

To better understand tissue generation in organ targets of autoimmune attack, it is instructive to examine the effects of these IP-modulating agents on different stem cells activity, effector T cell responses, NK cell responses, and the symptoms or markers of inflammatory diseases.

## Stem cell responses to IP-modulating agents in hair bulb

IFN-γ: IFN-γ activates the JAK–STAT signaling pathway (10, 11). Enhanced JAK–STAT activity suppresses hair follicle stem cell (HFSC) function *in vitro*, and STAT5 signaling promotes HFSC quiescence (12, 13). Conversely, inhibition of JAK kinases has been shown to restore epidermal stem cell function in aged skin, both *in vitro* and *in vivo* (14). Similarly, IFN-γ has been reported to inhibit hematopoietic stem cell (HSC) proliferation and differentiation (15) as well as the self-renewal capacity of these cells (16). In addition, IFN-γ rapidly inhibits hair elongation in cultured human anagen hair follicles and induces morphological features characteristic of catagen transformation (17). IFN-γ inhibits the proliferation and alters the differentiation of mesenchymal stem cells (18).

α-MSH: Treatment with α-MSH significantly accelerated hematopoietic recovery in irradiated mice (19).

IGF-1: Insulin-like growth factor 1 (IGF-1) is one of the most potent natural stimulators of cell growth and proliferation across multiple cell types in the body. IGF-1 can promote the proliferation and self-renewal of mesenchymal stem cells (MSCs) under specific *in vitro* conditions (20). IGF-1 is known to be the most potent anagen prolonging growth factor in HFs (21, 22). Activation of IGF-1 receptor (IGF-1R) signaling—whether through autocrine, paracrine, or inter-receptor cross-talk mechanisms—contributes to maintaining the self-renewal and pluripotent (or multipotent) capacities of stem cells both *in vitro* and *in vivo* (23). In squamous cell carcinoma, IGF-1 induced stem-like properties in carcinoma cells (24–26).

Tacrolimus: Tacrolimus stimulates hair growth when applied topically to the skin of normal mice, rats, and hamsters, but not when administered orally, even at doses that induce significant immunosuppression (27). As the authors indicated, **“**The hair growth stimulating effect of tacrolimus in normal animals may apparently be unrelated to its immunosuppressive effect**”**. Indeed, the immune system in normal animals is not expected to be highly active and is therefore less susceptible to immunosuppression. This suggests that topical tacrolimus may directly stimulate the proliferation of hair follicle stem or progenitor cells. As far as HSC are concerned, tacrolimus at clinically relevant concentrations enhanced clonogenesis of neutrophil progenitors and promoted their survival (28). No data was found on the effect of tacrolimus on the lymphogenic arm of hematogenesis at the level of stem and progenitor hematopoietic cells proliferation. Tacrolimus at low concentrations (2.4 x 10–^9^ M) did not affect the proliferation of adipose-derived stem cells, *in vitro*. However, at higher concentrations (2.4 x 10–^7^ M and 2.4 x 10–^6^ M), the proliferation rate increased in a dose-dependent manner (29). Another study reported that tacrolimus, up to 1.24 x 10–^4^ M, did not significantly impair MSC viability within gingiva-derived MSC spheroids cultured in osteogenic media (30).

TGF-β1: Exposure to TGF-β1 restores HF immune privilege following its collapse in the normal anagen hair bulb by downregulating MHC-I expression (9). TGF-β1 injection into back skin of mice promotes the transition of anagen hair into the catagen phase, a short intermediate stage that culminates in telogen—a phase which is fully quiescent most of the time. In addition, the number of proliferating follicle keratinocytes was reduced (31). In other words, TGF-β1 injected to rats reduced stem cell activities in the hair follicle and promoted the development of the early telogen phase characterized by quiescent stem cells. In contrast, TGF-β2 induces proliferation of HFSCs during the dormant telogen phase and promotes their entry into the tissue-regenerating anagen phase (32). In the bone marrow (BM), TGF-β1 is a potent inducer of HSC quiescence (33). However, this effect appears to be concentration-dependent: at high levels, TGF-β1 enforces quiescence, whereas at lower concentrations it may stimulate stem and progenitor cell proliferation (34). Within the tumor microenvironment (TME) of carcinomas, TGF-β1 drives epithelial cell to acquire properties of cancer stem cells (35).

## T and NK cell responses to IP-modulating agents in hair bulb

IFN-γ: Both *in vitro* and *in vivo*, autocrine IFN-γ produced by CD8^+^ T cells enhances their motility and promotes the killing of primary target keratinocytes (36). CD8^+^ T cells directly sense IFN-γ, which modulates T cell receptor (TCR) avidity and differentiation during infection. By supporting the expansion of low-avidity T cells, IFN-γ counterbalances the dominance of high-avidity clones during the primary response. At the same time, it promotes the long-term survival of high-avidity T cells within the memory pool, resulting in lower average avidity and suboptimal immunity during the primary response, but enhanced avidity and improved protection during recurrent infection (37). T cell reaction to IFN-γ is highly disease- or condition-related. In the setting of cancer, INF-γ inhibits Tregs, Th2 and Th17 differentiation and function but promotes CD8+ T and Th1 cell function (38). In experimental autoimmune encephalomyelitis (EAE) model and *in vitro*, IFN-γ induced the conversion of CD4+ CD25– T cells to CD4+ Tregs (39). Absence of IFN-γ receptor signaling attenuated graft-versus-host disease (GvHD) by enhancing Tregs expansion and facilitating their conversion (40). In a mouse model of minor histocompatibility mismatched corneal allografts, IFN-γ blocked Tregs and abolished immune privilege (41). The effect of IFN-γ on CD4+T cells is pleiotropic (42). IFN-γ has been reported to promote the accumulation, activation, and anti-cancer cytotoxicity of NK cells (43).

α-MSH: α-MSH, through its interaction with MC-1R, promotes the generation of tolerogenic dendritic cells capable of expanding Tregs both *in vitro* and *in vivo*. Importantly, these α-MSH-induced Tregs were functionally active, effectively suppressing cutaneous contact hypersensitivity and ongoing psoriasis-like skin inflammation in murine models (44). Additionally, a separate study demonstrated that α-MSH can directly convert effector T cells into Tregs (45). α-MSH also plays a key role in promoting the development of cytotoxic CD8+ T cells in both mice and humans (46). No data on the effect of α-MSH on NK cells could be found.

IGF-1: Recombinant human insulin-like growth factor 1 (IGF-1) stimulates the proliferation of both human and mouse Tregs *in vitro* and, when delivered systemically via continuous minipump, enhances their expansion (47). In contrast, IGF-1 increases IL-17 production by Th17 cells (48), and IGFs overall promote Th17 differentiation at the expense of Treg development. In the EAE model of multiple sclerosis, mice lacking IGF-1R specifically in T cells showed impaired disease progression (49). Four weeks of IGF-1 treatment in C57BL/6 mice led to significant increases in peripheral CD4+ and CD8+ naïve cell populations, as well as in total CD4 and CD8 T-cell receptor excision circle (TREC) content (50). It was also shown that IGF-1R signaling contributes to T cell dependent inflammation in arthritis (51). The effect of IGF-1 on NK cells is context dependent (52).

Tacrolimus: Calcineurin inhibitors, such as tacrolimus, inhibit the T-cell receptor-induced translocation of nuclear factor of activated T cells (NFAT) into the nucleus, thereby blocking Teff function and IL-2 transcription (53). At the same time, calcineurin inhibitors markedly disrupt Treg homeostasis and have a negative impact on the most highly immunosuppressive Treg subsets (54, 55). Calcineurin inhibitors have been reported to have a minimal impact on the cytolytic activity of NK cells *in vitro* (56).

TGF-β1: At low concentrations, TGF-β1 synergizes with IL-6 and IL-21 to promote IL-23R expression, favoring Th17 cell differentiation. This effect increases with TGF-β1 level and peaks at a specific TGF-β1 level. TGF-β1 concentrations above this level suppress IL-23R expression and promote the differentiation of Foxp3^+^ Treg cells (57, 58). Mice with T cell-specific deletion of TGF-β receptor II develop lethal inflammation associated with T cell activation and differentiation (59). *In vitro*, TGF-β1 suppressed the activation of naïve CD8^+^ T cells and reduced IFN-γ production by activated CD8^+^ T cells. In contrast, memory CD8^+^ T cells stimulated in the presence of TGF-β1 exhibited increased survival and produced higher levels of IL-17 alongside IFN-γ (60). However, it was also found that upon CD8^+^ T cell reactivation, TGF-β1 suppresses Granzyme B expression in an inverse relationship to the strength of TCR or proinflammatory signaling. In contrast, even high concentrations of TGF-β1 had only a modest effect on IFN-γ expression, regardless of whether the reactivation signals were weak or strong (61). An excellent review by Moreau et al. (62) describes the complexity of TGF-β1 signaling in Treg development and function. As clearly demonstrated, the effects of TGF-β1 on T cell activity are highly dependent on both its concentration and the cellular context. However, generally, high levels of TGF-β1 suppress T cell mediated immunity. For decades TGF-β1 was reported to inhibit the activation and functions of NK cells. However, contrasting evidence has emerged more recently (63). It seems that this effect is context-dependent.

## The effect of IP-modulating agents in hair bulb on various inflammatory conditions

IFN-γ: Elevated serum IFN-γ levels and markers of IFN-γ signaling in peripheral blood mononuclear cells (PBMCs) and kidneys have been reported in patients with various systemic AIDs, particularly systemic lupus erythematosus (SLE) (64). In numerous animal models of both spontaneous and induced systemic autoimmunity, disruption of the IFN-γ pathway has been shown to reduce disease severity (64). However, AMG-811, an anti-IFN-γ antibody, failed to demonstrate clinical improvement in a small cohort of 20 patients with discoid SLE (65). Similarly, Fontolizumab, a humanized anti-IFN-γ antibody, failed to elicit a strong clinical response in a Phase 2 trial of patients with moderate-to-severe Crohn**’**s disease (66). It was demonstrated that IFN-γ, can suppress arthritic inflammation in rats, and also contribute to resistance against arthritis (67). Although IFN-γ is traditionally known as a proinflammatory cytokine, it has been shown that IFN-γ cooperates with TNF-α to regulate the transition of macrophages from pro- to anti-inflammatory states (68). In experimental *Staphylococcus aureus* infection, IFN-γ conferred protection against septicemia but promoted the development of septic arthritis (69). In the tumor microenvironment, IFN-γ consistently orchestrates both pro-tumorigenic and antitumor immunity (70).

α-MSH: Treatment of BALB/cAn mice with pristane-induced lupus using the synthetic α-MSH analog NDP-MSH reduced arthritis scores by 70% and significantly decreased IgG1 and IgG2a levels, as well as the incidence of antinuclear antibodies (ANA). In the glomerulus, NDP–MSH treatment led to a 50% reduction in cellularity, accompanied by decreased IgG deposition and lower expression levels of α-smooth muscle actin (α-SMA), nitric oxide synthase (iNOS), and corticotrophin-releasing factor (CRF), marker of fibrosis, inflammation and renal damage (71). The systemic injection of α-MSH at the onset of paralysis in EAE was found extremely effective in diminishing the severity and tempo of EAE in SJL mice (72). In arthritic rats, peripheral α-MSH treatment demonstrated an anti-cachectic action increasing food intake and decreasing muscle wasting (73). The MC1r receptor agonists PL-8177 and PL-8331 exhibited actions similar to those of α-MSH in preventing and reversing intestinal and ocular inflammation in preclinical disease models (74).

IGF-1: IFG-1 may exert either pro- or anti-inflammatory effects in AIDs, depending on the target organs involved. IGF-1 halts autoimmune disease progression in mouse models of type 1 diabetes (T1D) and multiple sclerosis (MS) ***in vivo*** (47). A meta-analysis of 27 rheumatoid arthritis clinical studies revealed that decreased serum IGF-1 levels were closely associated with the development of rheumatoid arthritis (RA) (75), although the data varied considerably among studies. In juvenile idiopathic arthritis (JIA), a similar trend of reduced serum IGF-1 or Insulin-Like Growth Factor Binding Protein 3 (IGFBP-3) levels in patients compared to controls was observed. Notably, this trend was much more consistent, appearing in 8 out of 9 studies included in the review (76). A review by Zatorski et al. (77) concluded that IGF-1 exhibits anti-inflammatory properties in IBD and may be a promising therapeutic option for its treatment. In contrast, most studies involving patients with systemic lupus erythematosus (SLE) and systemic sclerosis (SSc) have reported increased serum levels of IGF-1 or IGFBP-3 (76). As for infections, it was reported that IGF-1 levels might contribute to the inflammatory response persistence and delayed lesion healing in human cutaneous leishmaniasis and the anemia development in visceral leishmaniasis (78). αVβ3 integrin is a coreceptor that is highly expressed by inflamed vascular endothelial and smooth muscle cells. αVβ3 antagonists block IGF-1-stimulated cell growth. A pig model of atherosclerosis demonstrated lesion size reduction by 48% following a 3-week infusion of αVβ3 antagonists. Multiple antibodies directed against the human IGF receptor have shown potent activity in cancer xenograft models (79). However, the outcomes of clinical trials investigating the use of anti-IGF-1 antibodies in cancer patients are conflicting (80).

Tacrolimus: Tacrolimus is widely used as an immunosuppressive agent, with diverse clinical applications. It is approved by multiple health authorities as a prophylactic therapy in hematopoietic stem cell transplantation to prevent GvHD (81). In South Korea, tacrolimus has also been approved for the treatment of rheumatoid arthritis (RA), lupus nephritis (LN), and myasthenia gravis (MYG) (82). Topical formulations are approved in many countries for moderate to severe atopic dermatitis (AD), and strong clinical evidence supports the broader dermatologic use of topical calcineurin inhibitors such as tacrolimus. These include conditions like vitiligo, psoriasis, seborrheic dermatitis, chronic hand dermatitis, contact dermatitis, oral lichen planus, lichen sclerosus, morphea, and cutaneous lupus erythematosus (83). Tacrolimus has also shown potential in the management of IBD, administered either systemically or topically (84). In pediatric nephrotic syndrome, results from 19 clinical trials reported remission rates ranging from 52.6% to 97.6% following tacrolimus treatment (85). However, topical tacrolimus has not been shown to be effective in alopecia areata (86).

TGF-β1: TGF-β1 plays dual roles in AIDs, inflammatory conditions, infections, and cancer:

*AIDs*: In lupus-prone mice, TGF-β1 expression is reduced in lymphoid tissues, and both TGF-β1 and TGF-β1–producing T cells suppress autoantibody production. In contrast, as these mice age and develop progressive organ damage and fibrosis, expression of TGF-β1 protein and mRNA, and signaling proteins increases in kidneys which are the target organs of lupus related autoimmunity. Thus, while TGF-β1 may offer protection against autoimmunity and inflammation, its sustained upregulation can potentially worsen the progression of inflammatory diseases (87). TGF-β1–knockout mice develop inflammatory conditions resembling AIDs, and even cell type–specific disruption of TGF-β signaling can trigger various AIDs in mice. While many AIDs are associated with dysregulated TGF-β signaling, the correlation with TGF-β levels can be either positive or negative (88).

*Inflammatory conditions*: TGF-β signaling exhibits a dual role in chicken embryos, providing protection in cases of intrauterine inflammation but exerting harmful effects during cranial neural crest development (89).

*Infections*: In leishmaniasis, TGF-β signaling plays opposing roles in immunity and pathogenesis. On one hand, it modulates the immune response to support Leishmania persistence and pathology; on the other, in combination with IL-6, TGF-β1 drives Th17 differentiation, thereby increasing tissue inflammation in infected lesions (90).

*Cancer*: In early-stage solid tumors, TGF-β1 suppresses tumor growth, whereas in advanced disease, as TGF-β1 accumulate in the tumor microenvironment (TME), it promotes tumor progression and metastasis by inhibiting anti-tumor immunity—effectively inducing immune privilege within the tumor microenvironment (TME) (91).

**Table 1** summarizes the effects of the agents modulating hair follicle immune privilege on stem cell activity, T cell and NK cell function and on various inflammatory conditions.

**Table 1:** The effect of agents modulating MHC-I expression in hair bulb on stem cell activities, T cell and NK cell function, and on various inflammatory conditions and cancer.

Agent	Effect on MHC-I- expression in HF	Effect on HFSCs activities	Effect on HSC MSC or CSC activities	Effect on T cell function	Effect on NK cell function	Effect on inflammatory conditions and cancer
IFN-γ	Upregulation (7, 9)	Drives quiescence in HFSCs (13), and inhibits hair elongation in cultured HF (17)	Inhibits HSC proliferation, differentiation and self-renewal (15, 16). Inhibits the proliferation and alters the differentiation of MSCs (18).	T cell reaction to IFN-γ is highly disease- or condition-related (36–41)	Promote anti-cancer NK cell activity (43)	Pathogenic in animal models of systemic autoimmunity (64). However, clinical anti-IFN-γ treatment does not seem to improve either SLE (65) or Chron’s disease (66) (but cohorts were small). Pleiotropic role in experimental arthritis (67) Plays pleiotropic role in infection (69) and cancer (70).
α-MSH	Downregulation (9)	NA	Upregulates the proliferation of HSCs and accelerates hematopoietic recovery (19)	α-MSH converted CD4+ T cells into Tregs (45) and expanded functionally active Tregs (44). At the same time, α-MSH induced the development of CD8+ T cells (46).	NA	Positive effect in pre-clinical models of SLE (71), EAE (72), arthritis (73), and IBD (74)
IGF-1	Downregulation (9)	a potent anagen prolonging growth factor (21, 22)	Promotes proliferation and self-renewal of many stem cells (20, 23). Induces stem-like properties in carcinoma cells (24–26)	Stimulates Tregs proliferation (47) but also IL-17 production by CD4 T cells. Mice lacking IGF-1R specifically in T cells exhibited impaired disease progression in EAE model [49 DiToro]. Peripheral CD4 and CD8+ T cells populations increased in mice following IGF-1 infusion (50). IGF-1R signalling contributes to T cell dependent inflammation in arthritis (51).	The effect on NK cell function is context-dependent (52)	Positive effect in EAE and preclinical T1D (47), and IBD (77). Negative effect in human leishmaniasis (78). Inhibition of IGF-1 activity improved atherosclerosis in a pig model (79).
Tacrolimus	Downregulation (9)	Tacrolimus stimulates hair growth when applied topically to normal animal skin (27). This indicates induction of stem and progenitor cell activities since immune suppression in normal skin is unlikely.	Tacrolimus does not affect the proliferation of adipose-derived stem cells at low concentrations; however, it accelerates their proliferation at high concentrations (29). Tacrolimus does not impair viability of MSC spheroids in osteogenic media (30)	Blocks T effector function but also impairs Treg activities (53–55)	A minimal impact on the cytolytic activity of NK cells *in vitro* (56)	Approved oral use in GvHD, RA, LN, GYD (82) and topical use in AD. Topical off-label use in many dermatologic conditions (83). Also found effective in IBD (84) and NS (85). Ineffective in alopecia areata (86)
TGF-β	Downregulation by TGF- β1 (9). Downregulation by TGF-β2 (150) Berglund 2017, (151) Berglund 2020]	TGF-β1 promotes quiescence in HFSCs (31). TGF-β2 activates quiescent HFSCs to tissue regenerative mode (32)	TGF-β1 induces quiescence in HSCs at high concentrations, whereas low concentrations stimulate stem/progenitor cells proliferation (34). TGF-β1 converts epithelial cells to CSC-like cells (35)	Effect on T cells is highly plieotropic and depends on concentration and context. However, high TGF- β1 levels generally suppress T cell activities (57, 59–62)	The effect is context-dependent (63)	TGFβ1 Plays pleiotropic role in cancer, inflammation, infectious diseases, and AIDs (87, 89–91)

IFN-γ, interferon γ; α-MSH, α-melanocyte stimulating hormone; IGF-1, insulin-like growth factor 1; TGF-β, transforming growth factor β; MHC-I, major histocompatibility complex class 1; MHC-II, major histocompatibility complex class 2. HSC, hematopoietic stem cell; MSC, mesenchymal stem cell; CSC, cancer stem cells; NA, not available.

**Table 1** demonstrates that: (a) HFSCs, HSCs, and MSCs generally respond in a comparable manner to all five listed agents; and (b) IFN-γ, which induces HF-IP collapse, also drives quiescence in HFSCs and inhibits HSC and MSC proliferation, whereas agents that restore HF-IP—except for TGF-β1—tend to promote stem cell self-renewal and/or proliferation. The implications of these observations will be explored in the following sections.

## Enhancement of stem cell activity by α-MSH, IGF-1, and tacrolimus confers beneficial effects in autoimmune diseases

**Table 1** shows that α-MSH, IGF-1, and tacrolimus alleviate symptoms of various AIDs in both animal models and humans. However, IGF-1 has also shown negative effects in certain inflammatory conditions not driven by autoimmunity. Autoimmune attacks may engage, as dominant effectors, CD8+ T cells (e.g. T1D, vitiligo, AA (6, 92, 93)), CD4+ T cells (e.g. MS, SLE, RA, IBD, EAE, autoimmune thyroid diseases, psoriasis (94)), NK cells (e.g. AA, SLE, RA, MS, psoriasis, T1D (95, 96)) or autoantibodies (e.g. SLE, T1D, psoriasis, Grave**’**s disease, IBD, MS (97)). However secondary effectors are frequently involved as well. Inspection of **Table 1** reveals that both α-MSH and IGF-1, which promote stem cell proliferation and renewal, also promote Tregs proliferation. At the same time, both promote CD8+ T cell function while IGF-1 also promote CD4+ T cell expansion and function. Tacrolimus, also, enhances stem cell proliferation but suppresses both Tregs and CD8+ T cells function.

Equation 1 is useful in interpreting these observations. As previously noted, a constitutive positive value of *R*, the net generation rate, reflects improvement in autoimmune disease symptoms, whereas a prolonged negative value corresponds to symptom exacerbation. Agents that raise *R* value are considered beneficial, while those that lower it are deemed detrimental.

α-MSH and IGF-1, by enhancing stem cell activity and supporting tissue regeneration, contribute to an increase in *Rreg* in Equation 1. However, because both agents stimulate Treg proliferation while also enhancing Teff activity—with opposing effects on immune attack—their net impact on *Rdes* is not immediately evident. Given that Treg function is impaired in most AIDs (98), the influence on Teff cells is likely to be more consequential. Given that both agents have been shown to alleviate symptoms in several AIDs, it is likely that the regenerative enhancement through stem cell activation outweighs the cytotoxic activation they also promote. This discussion excludes the effects of both agents on NK cells, as relevant data are either unavailable or context-dependent and difficult to predict.

Tacrolimus stimulates stem cell activity and impairs effector T cell function, both of which contribute to an increase in the tissue**’**s net generation rate. Its inhibitory effect on Tregs function is less important, since this function is impaired in many AIDs (98). The regenerative and immunosuppressive effects of tacrolimus explain its clinical benefit in various autoimmune diseases.

## Immune privilege is not an intrinsic property of quiescent stem cells but depends on the mechanism inducing their quiescence

As seen in **Table 1**, both IFN-γ and TGF-β1 downregulate stem cell activity and induce quiescence. IFN-γ activates Jak-Stat signaling (10), which suppresses epidermal stem cell functions (14) and induces quiescence in HFSCs (99). TGF-β1, on the other hand, has been shown to induce quiescence in tumor-propagating cancer cells through activation of TGF-β/SMAD signaling, which directly regulates cell cycle gene transcription to elicit a reversible G1 arrest (100). Another pathway that downregulates hair regeneration is the inhibition of Wnt/β-catenin signaling by bone morphogenetic protein (BMP) during telogen (13, 101). Thus, conventional telogen can be divided into two phases: one driven by high BMP signaling, which impedes hair regeneration, and another driven by low BMP signaling, which promotes intensive hair regeneration (101). The three mechanisms that induce quiescence in stem cells and tumor-propagating cells differ in their effects on MHC-I expression. TGF-β1 downregulates MHC-I expression in hair epithelia, whereas IFN-γ upregulates it (9). Activation of the Wnt pathway has been associated with decreased MHC-I expression in the tumor microenvironment. If the same holds true in the hair follicle, then BMP, which inhibits Wnt signaling, would be expected to upregulate MHC-I expression in this context. It therefore appears that low MHC-I expression in the HF (i.e., immune privilege) is not an inherent property of the quiescent state, but rather depends on the specific pathway that induces quiescence. *This work hypothesizes that constitutive exposure to high levels of TGF-β1 can induce long-lasting quiescence that persists even after TGF-β1 withdrawal.* For example, repeated intradermal injections of 0.3 µg recombinant human TGF-β1, administered twice daily for three consecutive days into the tail region of depilated back skin of C57BL/6 adolescent mice in anagen, triggered premature catagen development associated with reduced follicular proliferation (31). These follicles subsequently entered early telogen, characterized by quiescent HFSCs, demonstrating a sustained effect of repeated TGF-β1 exposure. A study of the kinetics of MHC-I downregulation following exposure of bone marrow–derived MSCs (from humans and horses) to TGF-β for at least 72 hours supports this hypothesis. TGF-β1, TGF-β2, and TGF-β3 were all similarly effective at downregulating constitutive MHC I expression. After treatment, a slight but non-significant decrease in MHC-I surface expression was observed at 24 hours, followed by significant downregulation at 48 and 72 hours (the latest time point assessed). In parallel, RNA sequencing of MSCs revealed significant downregulation of two antigen processing and presentation genes, B2M and ERAP1, at 48 and 72 hours post-treatment (102). These results indicate that TGF-β1 exerts a persistent effect on MHC-I surface expression in stem cells, lasting well beyond its rapid clearance from circulation (TGF-β1active form half-life ~2 minutes (103)).

It has been demonstrated that quiescent HFSCs are protected from T cell–mediated killing even after a marked reduction in Tregs, achieved by injecting an anti-CD25 antibody into mice (104). In addition, Jedi T cells (GFP-specific CD8 T cells) cultured with GFP^+^ HFSCs did not proliferate, indicating that HFSCs fail to activate T cells even when they present an antigen recognized by them (104). Based on these findings, it was concluded that **“**even though Tregs around the bulge may be immunosuppressive, they were not the sole mechanism of protection**”** (105). This conclusion assumes that Tregs exert only a short-lived effect. However, prolonged TGF-β1 secretion by Tregs prior to their depletion, or before hair follicles were transferred into culture, may have induced a long-lasting effect on MHC-I expression and, consequently, on immune privilege.

Importantly, although long-lasting, this effect is not irreversible: it can be disrupted by agents such as IFN-γ, which upregulate MHC-I expression and trigger collapse of immune privilege.

## The immune privilege paradox of anagen hair follicles exposed to IFN-γ

In their seminal work, Agudo et al. (104) demonstrated that quiescent stem cells in the hair follicle and muscle are resistant to T cell–mediated killing due to downregulation of the MHC-I transactivator NLRC5. The authors showed that quiescent stem cells in the hair follicle bulge are protected during anagen, even in the presence of high numbers of activated T cells, whereas other stem cells and differentiated cells within the HF outside the bulge are susceptible to T cell attack. This immune-evasive mechanism preserves a reservoir of stem cells capable of regenerating damaged hair follicle cells following immune injury.

According to Ito et al. (9), IFN-γ induces aberrant MHC-I expression in the normally MHC-I-negative anagen hair matrix epithelium, thereby increasing the bulb**’**s susceptibility to CD8+ T cell attack and promoting the collapse of immune privilege. However, IFN-γ also induces quiescence in HFSCs (13), which, as shown by Agudo et al. (104), leads to MHC-I downregulation and imparts resistance to CD8+ T cell-mediated assault. These findings appear to be contradictory.

I propose a potential explanation for this apparent paradox. IFN-γ renders the hair bulb susceptible to cytotoxic attacks that destroy anagen epithelial cells, as well as stem cells in the outer root sheath during anagen (106), while sparing the stem cells residing in the bulge region (105)—a pattern reminiscent of the immune-mediated follicular damage observed in alopecia areata, where bulge stem cells remain intact (4, 5). This results in hair shedding. Simultaneously, hair follicle stem cells in the bulge become arrested in the G_0_ phase and enter a quiescent state, thereby halting new hair growth. Although quiescence promotes immune privilege in the bulge, it also inhibits hair regeneration. In short, following anagen hair exposure to IFN-γ, MHC-I is upregulated in the outer root sheath but downregulated in the bulge. The following paragraph proposes a potential mechanism to explain this disparity in MHC-I expression between the bulge and non-bulge regions.

## Why the bulge remains protected from cytotoxic attack despite immune privilege collapse in the hair bulb during alopecia areata

**Figure 1:**
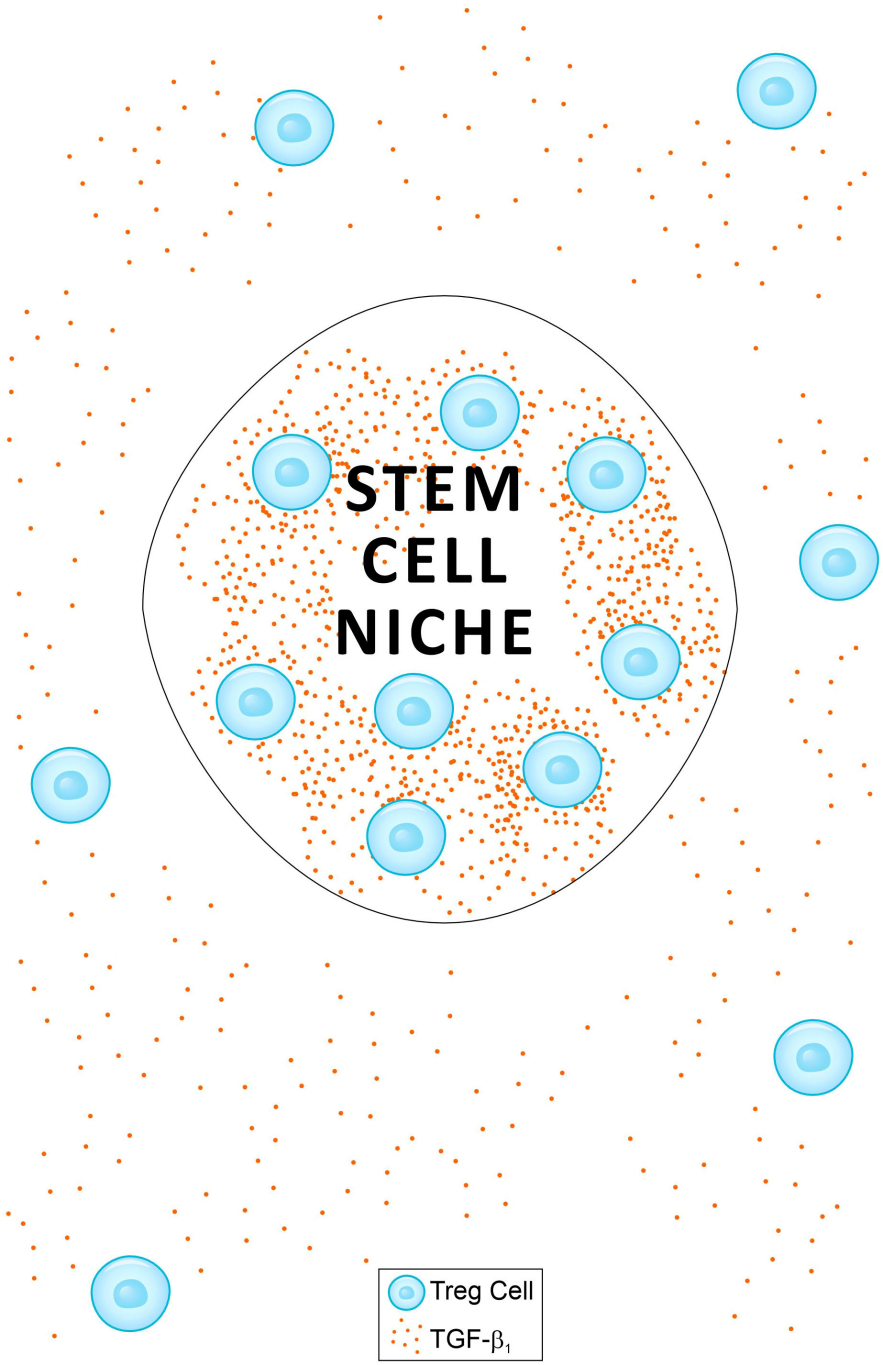
A schematic presentation of a model proposed as an explanation for two phenomena: **(a)** the immune privilege paradox of anagen hair follicles (HF) when exposed to IFN-γ (see main text); and **(b)** the protection of the hair bulge from cytotoxic attack during alopecia areata. A relatively high frequency of regulatory T cells (Tregs) within the bulge creates a TGF-β1 gradient between areas inside and outside the bulge. The effect of TGF-β1 on stem cells is concentration-dependent, inducing quiescence at high levels and promoting activity at lower levels (34). This TGF-β1 gradient may induce quiescence of hair follicle stem cells (HFSCs) within the bulge while stimulating their proliferation and differentiation outside the bulge. This dual mechanism preserves the stem cell reservoir in the bulge from autoimmune attack (104), while supporting the generation of new cells required for tissue homeostasis outside the bulge. A similar model applies to hematopoietic stem cells (HSCs) in bone marrow niches.

It has been reported that during anagen, follicular stem cells in the outer root sheath become susceptible to CD8^+^ T cell attack and upregulate MHC-I expression (105). Regulatory T cells in the skin have been shown to preferentially localize to the HFSC niche, particularly during the telogen phase, but also during anagen (107, 108). Tregs are a major source of TGF-β1 in both animal and human tissues. I propose that the relatively high abundance of Tregs in the hair bulge creates a TGF-β1 concentration gradient between the bulge and the outer root sheath outside the bulge (**Figure 1**). The effect of TGF-β1 on HSCs has been shown to be concentration-dependent, inducing quiescence at high concentrations while promoting stem cell activity at lower levels (34). If this pleiotropic effect also applies to HFSCs, a TGF-β1 concentration gradient may induce quiescence of HFSCs within the bulge, while stimulating their proliferation and differentiation outside the bulge. In this model, TGF-β1 secreted locally by Tregs simultaneously maintains stem cell quiescence in the bulge and promotes renewal and differentiation in adjacent regions. This dual mechanism both preserves the stem cell reservoir in the bulge from autoimmune attack (104) while outside the bulge it supports the generation of new cells required for tissue homeostasis. In contrast, injection of TGF-β1 into the dorsal skin of rats reduced HFSC stem cell activity and promoted the transition from anagen to catagen (31). In this context, high concentrations of TGF-β1 both within and outside the bulge similarly impaired stem cell activity.

I propose that the TGF-β1 concentration gradient between the bulge and the surrounding hair follicle regions observed in healthy skin, persists during alopecia areata. Although the frequency of FoxP3+CD39+Treg cells is reduced in both the hair follicles and circulation of AA patients compared to healthy controls, no significant differences in the frequency of TGF-β1+Tregs were observed between the two groups (109). This TGF-β1 concentration gradient may help maintain bulge stem cell quiescence and protect the bulge from cytotoxic CD8^+^ T cell attacks during AA, while stem cells outside the bulge remain active and more susceptible to immune-mediated destruction.

## Effects of IFN-γ and TGF-β1 on hair follicle in normal hair and in alopecia areata

IFN-γ induces quiescence in active HFSCs, thereby halting hair growth. Indeed IFN- γ has shown to be a potent inducer of catagen-like changes in cultured human anagen hair follicles (17). Simultaneously, by upregulating MHC-I expression in hair bulb epithelial cells, IFN-γ heightens the follicle**’**s vulnerability to CD8+ T cell attack (9). However, in normal hair, CD8+ T cell attack of hair follicle is unlikely and follicular cell destruction is unlikely following exposure to IFN-γ. Therefore IFN-γ is expected to drive the normal anagen hair bulb to catagen-like phase, ***in vivo*** as well. In active AA, external supply of IFN-γ is not expected to generate any effect since HFSCs outside the bulge were probably destroyed following IP collapsed.

External high levels of TGF-β1 downregulates MHC-I expression in hair bulb epithelial cells (9), protecting the bulb from immune attack, while simultaneously inducing quiescence in active HFSCs (31). Since normal anagen hair epithelium demonstrates low MHC-I expression (9), and cytotoxic cells are not activated under normal conditions, downregulation of MHC-I would exert no effect on normal hair. On the other hand, external high dose of TGF-β1 is expected to drive growth arrest in normal hair due to the downregulation of HFSC activity outside the bulge, and drive anagen to catagen transition, as indeed reported in normal mice injected with TGF-β1 (31). As explained in the preceding section, the TGF-β1 gradient between regions inside and outside the bulge is maintained in alopecia areata. External TGF-β1 application in alopecia areata will restore immune privilege while inducing quiescence in HFSCs outside the bulge. This will prevent immune attack, but hair growth (remission) by external TGF-β1 is not expected in AA.

## Effects of IFN-γ and TGF-β1 on bone marrow under normal conditions, autoimmune diseases, and cancer

Similar to their accumulation in the hair follicle bulge, Tregs in the bone marrow localize along the bone endosteal surface, where HSC niches reside, providing immune privilege to these niches (110). As shown in **Table 1**, TGF-β1 induces quiescence in both HFSCs and HSCs. I therefore propose that HFSCs and HSCs are immune protected within their niches due to high local TGF-β1 concentrations, whereas lower concentrations outside the niches promote both stem cell activity and reduced protection against autoimmune attack. Both IFN-γ and high levels of TGF-β1 induce quiescence in HSCs outside the niches, resulting in low production of bone marrow (BM) cells. Although healthy BM is likely subject to immune surveillance for apoptotic cells, invading pathogens, and malignant transformation (111), its normal cells are generally neither targeted nor destroyed. However, following exposure to cytokines that alter HSC activity or survival, BM tissue can fluctuate between destruction and regeneration—paralleling the dynamics seen in the hair follicle.

In certain tissues targeted by autoimmune attacks, in addition to variations in cell number, variations of function also influence the likelihood of remission or relapse. For instance, fluctuations in insulin secretion rates by pancreatic β cells in Type 1 Diabetes or changes in thyroid hormone secretion by thyroid follicles in Hashimoto**’**s Thyroiditis can trigger symptom remission or relapse. In such a case, as proposed in earlier publication (1), Equation 1. should be replaced by:

(3) Ra= (Ra+)–(Ra −)

where *Ra* represents any tissue activity—such as a hormone secretion rate—that depends on both the number and the function of secreting cells. *Ra+* represents the increase in *Ra* by factors like tissue regeneration or activating agents, while *Ra-* indicates the decrease in *Ra* due to tissue destruction following autoantibody attack, or from impairment of cell function by deactivating hormones or toxic substances. Equation 3 can thus be viewed as a generalization of Equation 1, since cell generation rate is only one of the factors contributing to tissue activity.

Immune cells constitute a major component of the bone marrow, and their influence on AIDs, infections, and cancer depends not only on their abundance but also on their functional state, making Equation 3 suitable for representing bone marrow **“**tissue activity.**”** In this context, *Ra* in Equation 3 denotes the cytotoxic activity of bone marrow–resident immune cells—that is, the extent of target destruction, whether of tissue or pathogen, inflicted per unit time. The bone marrow–related *Ra* is a positive value. Hematopoietic stem cell renewal and proliferation, as well as factors that enhance the activity of bone marrow–resident immune cells, contribute to *Ra^+^*. Immune cell destruction within the bone marrow due to autoimmune attack (e.g., in autoimmune lymphocytopenia) or exposure to agents that reduce immune cell numbers and/or impair their function within bone marrow, contributes to *Ra^-^*.

High Treg numbers and elevated TGF-β1 levels within HSC bone marrow niches induce HSC quiescence and confer immune protection to the niche. In contrast, HSCs and differentiated immune cells located outside the niches are active and unprotected. These differentiated immune cells exhibit a baseline activity rate in healthy animals at homeostasis, designated here as *Ra(0).*

It is convenient to define *ΔRa*, as the difference between *Ra* and *Ra(0)*:

(4) ΔRa=Ra−Ra(0)

At homeostasis, *ΔRa* = 0 in healthy humans or animals. An increase in cytotoxic activity relative to *Ra(0)* corresponds to *ΔRa* > 0, whereas a decrease in activity corresponds to *ΔRa* < 0.

*The effect of TGF-β1 on BM*: In the hematopoietic system, low TGF-β1 concentrations (pg/mL) stimulate HSC proliferation, whereas higher concentrations (ng/mL) are inhibitory (34). Exposure of normal BM to externally high TGF-β1 levels causes HSCs outside the BM niches to enter dormancy, preventing the generation of new immune cells (resulting in a decrease in *Ra+*). High TGF-β1 levels also promote immune privilege. However, under normal conditions, IP exerts minimal effects on BM cells, as the BM experiences little immune attack. Nevertheless, internal immunosurveillance of BM has been reported in normal marrow, mediated by local CD8^+^ T cells and resident dendritic cells (DCs), which activate antigen-specific T cells, including CD8^+^ T cells, within the BM (112), as well as resident macrophages (113). High concentrations of TGF-β1 induce the production of Tregs (57), impair the function of activated CD8^+^ T cells (60), and promote anti-inflammatory M2 polarization of macrophages (114). Conversely, TGF-β1 can enhance the cytotoxicity of memory cytotoxic T cells (60) and increase Granzyme B expression upon reactivation of CD8^+^ T cells (61). The net effect of high TGF-β1 levels on *ΔRa* is therefore unclear—*ΔRa* may be positive or negative and is likely context dependent. Consistently, while a physiological chronic increase in TGF-β signaling has little impact on hematopoiesis, an additional acute insult with a potent innate immune activator can induce persistent, ineffective hematopoiesis resembling myelodysplastic BM failure (115).

*The effect of IFN-γ on BM*: If normal BM is exposed to IFN-γ, HSCs outside the niches will enter dormancy, preventing the generation of new immune cells. However, unlike TGF-β1, these HSCs are not expected to develop immune privilege—by analogy with hair follicles, where IFN-γ triggers immune privilege collapse in anagen follicles (9, 116). IFN-γ has been shown to stimulate dendritic cells *in vitro* (117) and macrophages *in vivo* (118), and may therefore activate BM-resident immune cells to attack the BM, reducing both the number and activity of progenitor immune cells. Collectively, these effects result in *ΔRa* < 0. Indeed, prolonged exposure to IFN-γ has been reported to induce BM failure and aplastic anemia (119). IFN-γ is also associated with hematopoietic suppression observed in patients with Fanconi anemia (120)and HIV (121).

Collectively, the hematopoietic system in healthy humans and animals exists in a steady state that can fluctuate under the influence of different cytokines. IFN-γ generally downregulates hematopoiesis and immune cell activity, whereas the effects of TGF-β1 are highly pleiotropic. High TGF-β1 concentrations within HSC niches protect HSCs from damage, while lower levels outside the niches allow HSCs in these areas to remain active. Both IFN-γ and high TGF-β1 levels induce quiescence in HSCs outside the niches. However, high TGF-β1 also promotes immune privilege outside the niches, whereas IFN-γ does not induce IP; instead, it enhances BM immunosurveillance and triggers attacks by resident immune cells.

## Pleiotropic effects of IFN-γ and TGF-β in autoimmune diseases, infections, and cancer

Indeed, IFN-γ and TGF-β both play dual roles in autoimmune diseases, infections and cancer (122). In AIDs, each can exert both pro-inflammatory and anti-inflammatory effects (87, 89, 123). Similarly, each cytokine may have opposing effects during the course of certain infections (69, 90). Within the tumor microenvironment (TME), both contribute to pro-tumorigenic activity as well as antitumor immunity (70, 91).

Several detailed examples illustrate these pleiotropic effects:

IFN-γ: The impact of IFN-γ on EAE depends on the timing of its expression in the CNS (124). Studies in transgenic mice with temporally regulated CNS expression of IFN-γ show that expression before EAE onset improves disease course and prevents oligodendrocyte loss, demyelination, and axonal degeneration (125). In contrast, IFN-γ expression during the recovery stage suppresses oligodendrocyte regeneration and remyelination within lesions (126). IFN-γ demonstrates a dual role in experimental *Staphylococcus aureus* infection by protecting against septicemia while promoting the development of septic arthritis (69). In cancer, tumors treated with low-dose IFN-γ acquired metastatic properties, whereas high-dose IFN-γ induced tumor regression (70).

TGF-β1: The effects of TGF-β1 are both concentration- and context-dependent. In lupus-prone mice (87) and in mice transgenic for an active form of TGF-β1 (127), low concentrations suppress the immune response, whereas accumulation of TGF-β in the kidneys exerts proinflammatory effects. Conversely, in cancer, low TGF-β concentrations inhibit tumor growth, while high concentrations suppress antitumor immunity, promoting tumor cell proliferation (91). TGF-β also exhibits opposing roles in immunity and pathogenesis during leishmaniasis (90).

## The contrasting effects of TGF-r superfamily isoforms on stem cell activities

The TGF-β superfamily is a diverse group of growth factors, including TGF-βs, BMPs, Activin, Inhibin, and Nodal. These ligands bind to specific cell surface receptors, triggering a complex signaling cascade. Once activated, these receptors phosphorylate and activate Smad proteins, which serve as the primary intracellular mediators of TGF-β superfamily signaling (128). Different members of this family exert contrasting effects on stem cell activity, reflecting the pleiotropic nature of this cytokine group. For example, as previously described, TGF-β1 suppresses HFSC activity (31), whereas TGF-β2 promotes HFSC proliferation (32). Bone morphogenetic protein 4 (BMP4) promotes HSC development both *in vitro* (129) and *in vivo* (130), whereas the effect of TGF-β1 on HSCs is biphasic and concentration-dependent (34). Activin A–induced signaling plays a critical role in hair follicle neogenesis (131), while TGF-β1 drives quiescence in HFSCs (31). BMP9 is among the most potent BMPs for inducing osteogenic differentiation. In contrast, another member of the TGF-β superfamily, Inhibin-α, has been shown to inhibit BMP9-induced osteogenic differentiation in MSCs (132). Thus, the pleiotropic character of the TGF-β superfamily is reflected in its diverse roles across different organs.

## The contrasting roles of IFN-γ and TGF-β1 in tissue homeostasis

High levels of TGF-β1 induce stem cell quiescence while simultaneously enhancing immune privilege and reducing immune attack. Consequently, elevated TGF-β1 supports the maintenance of tissue homeostasis and aids in restoring balance in tissues experiencing homeostatic disruption.

**Figure 2:**
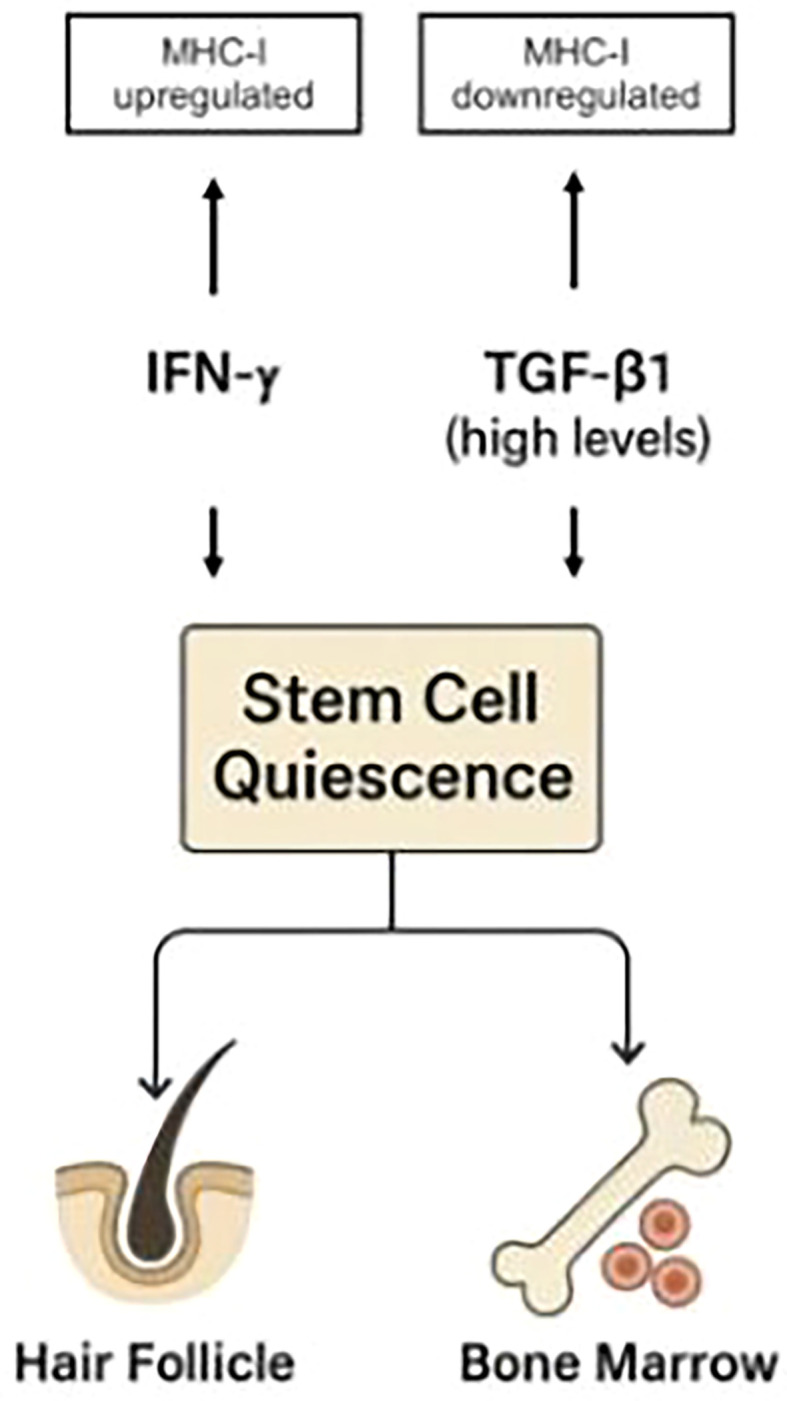
While both IFN-γ and high levels of TGF-β1 induce stem cell quiescence, they exert opposite effects on immune privilege: IFN-γ upregulates MHC-I expression, whereas TGF-β1 downregulates it. Similar mechanisms apply to hematopoietic stem cell niches in the bone marrow. This demonstrates that immune protection is not an inherent feature of quiescent stem cells, as previously proposed (104), but instead depends on the specific pathways that regulate quiescence. The TGF-β/Smad pathway not only induces quiescence in stem cells (100) but also drives MHC-I downregulation (9), thereby explaining the association between stem cell quiescence and immune privilege. In contrast, IFN-γ induces quiescence while upregulating MHC-I expression.

Like high levels of TGF-β1, IFN-γ also induces stem cell quiescence (13, 15, 16). However, unlike TGF-β1, it disrupts immune privilege and heightens tissue immune surveillance. In the immune-privileged, anagen-phase hair follicle with active stem cells, external IFN-γ can trigger catagen (17)—representing a new equilibrium state. In a hair follicle under immune attack, as in alopecia areata, IFN-γ will cause immune-privilege collapse and disrupted homeostasis. In the bone marrow, which is normally under immune surveillance, IFN-γ will promote homeostatic imbalance and tissue damage (119). **Figure 2** schematically illustrates these effects.

Thus, elevated TGF-β1 supports tissue homeostasis, while IFN-γ acts to disrupt it. This generalizes the observation that IFN-γ upregulates MHC-I expression in HF epithelia, whereas TGF-β1 downregulates it (9).

## Effects of IFN-γ and TGF-β1 on bone marrow contributing to their pleiotropic nature

**Figure 3:**
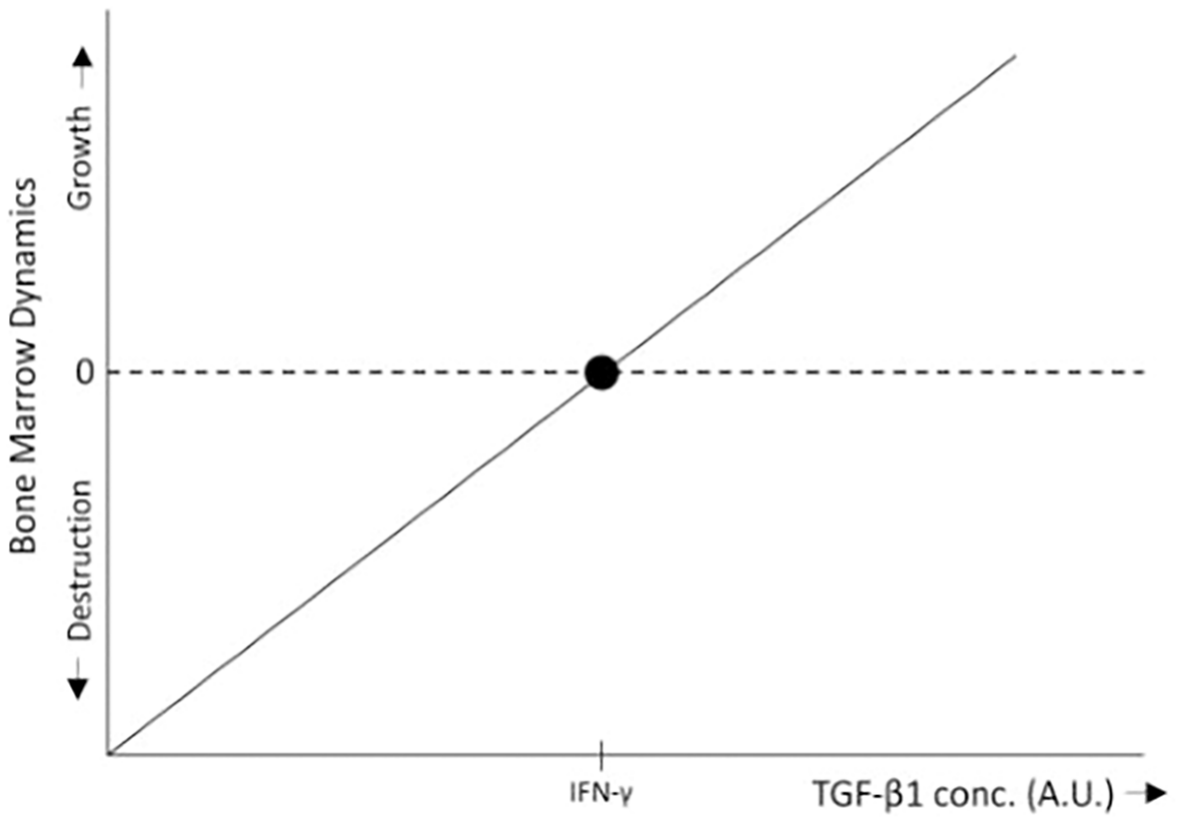
In the bone marrow, low levels of TGF-β1 promote HSC proliferation (34), leading to bone marrow growth, whereas IFN-γ inhibits HSC proliferation, differentiation, and self-renewal (15, 16), thereby promoting bone marrow destruction through immune surveillance. Within the range of low TGF-β1 concentrations, when TGF-β1 levels exceed a certain threshold relative to IFN-γ, bone marrow growth is induced. Conversely, when TGF-β1 levels fall below this threshold, the inhibitory effects of IFN-γ predominate, resulting in bone marrow destruction. For simplicity, the schematic representation assumes a linear relationship between bone marrow growth and TGF-β1 levels.

**Figure 4:**
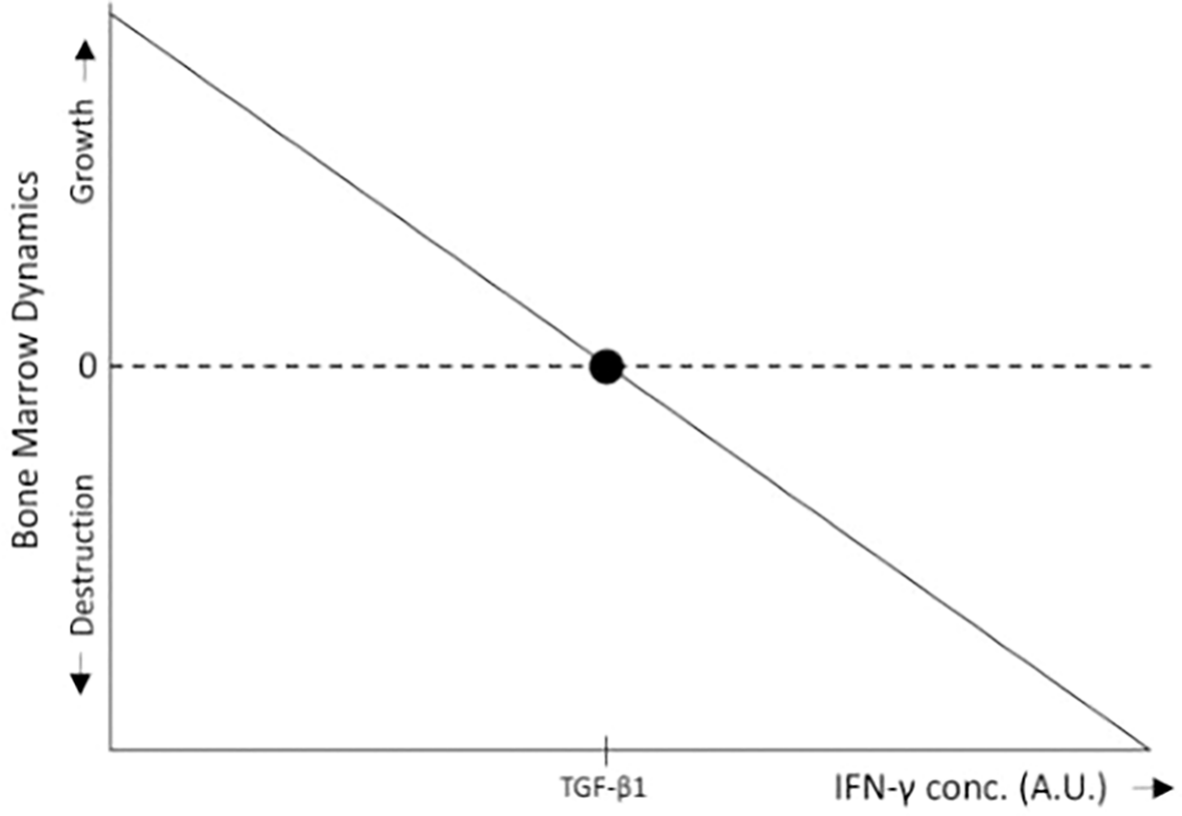
In the bone marrow, IFN-γ inhibits HSC proliferation, differentiation, and self-renewal (15, 16), thereby promoting bone marrow destruction through immune surveillance, whereas low levels of TGF-β1 stimulate HSC proliferation (34), leading to bone marrow growth. Within the range of low TGF-β1 concentrations, when IFN-γ levels fall below a certain threshold relative to TGF-β1, the proliferative effects of TGF-β1 predominate, resulting in bone marrow expansion. Conversely, when IFN-γ levels exceed this threshold, bone marrow destruction occurs. For simplicity, the schematic representation assumes a linear relationship between bone marrow growth and IFN-γ levels.

Consider a tissue at homeostasis exposed to two cytokines: A, which promotes tissue expansion (e.g., a growth factor), and B, which drives tissue destruction (e.g., an immune activator). With B constant, high levels of A favor tissue growth, whereas low levels of A relative to B lead to immune-mediated tissue destruction. In the absence of B, or when B is inhibited (as in immune-privileged tissues), both low and high levels of A promote tissue expansion, albeit at different rates. Agent A thus exemplifies a pleiotropic factor with both a concentration-dependent effect (high vs. low levels) and a context-dependent effect (presence or absence of B). In the bone marrow, TGF-β1 (in the low level region) serves as an example of A, while IFN-γ represents B (**Figures 3**, **4**).

Another mechanism that may contribute to the pleiotropic effects of cytokines involves circulating cytokines that influence stem cell activity both in a specific organ and in the bone marrow. IFN-γ, for example, induces quiescence in both HFSCs and HSCs. Although IFN-γ increases MHC-I expression in the hair follicle epithelium and NKG2D expression in CD56^+^ NK cells (9, 116), it is not expected to trigger an immune attack on the HF under normal conditions, as CD8+T cells and NK cells remain inactive in such contexts. Consistently, mice injected with IFN-γ showed no hair loss (133). Conversely, IFN-γ enhances BM immune surveillance and promotes the elimination of immune cells in the BM in both healthy individuals and AA patients. The resulting reduction in cytotoxic progenitor cells and NK cells counteracts the IFN-γ–induced decrease in hair follicle IP. The relative contribution of these opposing effects may depend on other cytokines and is relevant to AA pathogenesis. Thus, the impact of IFN-γ on AA is likely context-dependent. In agreement, among 16 AA patients treated with anti–IFN-γ antibodies, eight of nine with patchy, progressive hair loss responded well, whereas treatment success was very limited in the five patients with complete baldness (134). Moreover, although serum IFN-γ levels correlated with AA activity, no association was found between serum IFN-γ and disease severity (135).

To summarize, two mechanisms are proposed to underlie the pleiotropic effects of IFN-γ and TGF-β1. The first arises from their opposing effects on BM activity, with IFN-γ and high levels of TGF-β1 exerting contrasting influences. The second stems from the opposing effects of IFN-γ on the intensity of immune attack in the BM versus the target tissue during autoimmune disease.

## The role of stem cells in flares and remissions of autoimmune diseases

Symptoms and flares of AIDs are initiated by either internal or external causes. Internal causes include epitope spreading, reduced immune regulation, and an increased inflammatory response (136) while external causes include viral infections, low exposure to sunlight (137), and stress (138).

Flares in AIDs are characterized by a negative *ΔM* value in Equation 2 or a negative *ΔRa* in Equation 4, whereas remissions occur when *ΔM* or *ΔRa* becomes positive. As discussed above, cytokines such as IFN-γ, TGF-β, α-MSH, and IGF-1 influence stem cell activity in AIDs target tissues as well as in bone marrow. In addition, these cytokines may directly modulate the activity of autoreactive immune cells and affect bone marrow surveillance. Some exhibit contrasting effects on these processes. The specific combination of cytokines presence, along with the presence or absence of other modulatory cytokines, will determine whether *ΔM* in Equation 2 and *ΔRa* in Equation 4 are positive or negative, thereby driving either improvement or worsening of disease symptoms. Consequently, different cytokine profiles, shaped by both internal and external factors, can trigger flares or induce remissions in autoimmune diseases.

**Figure 5:**
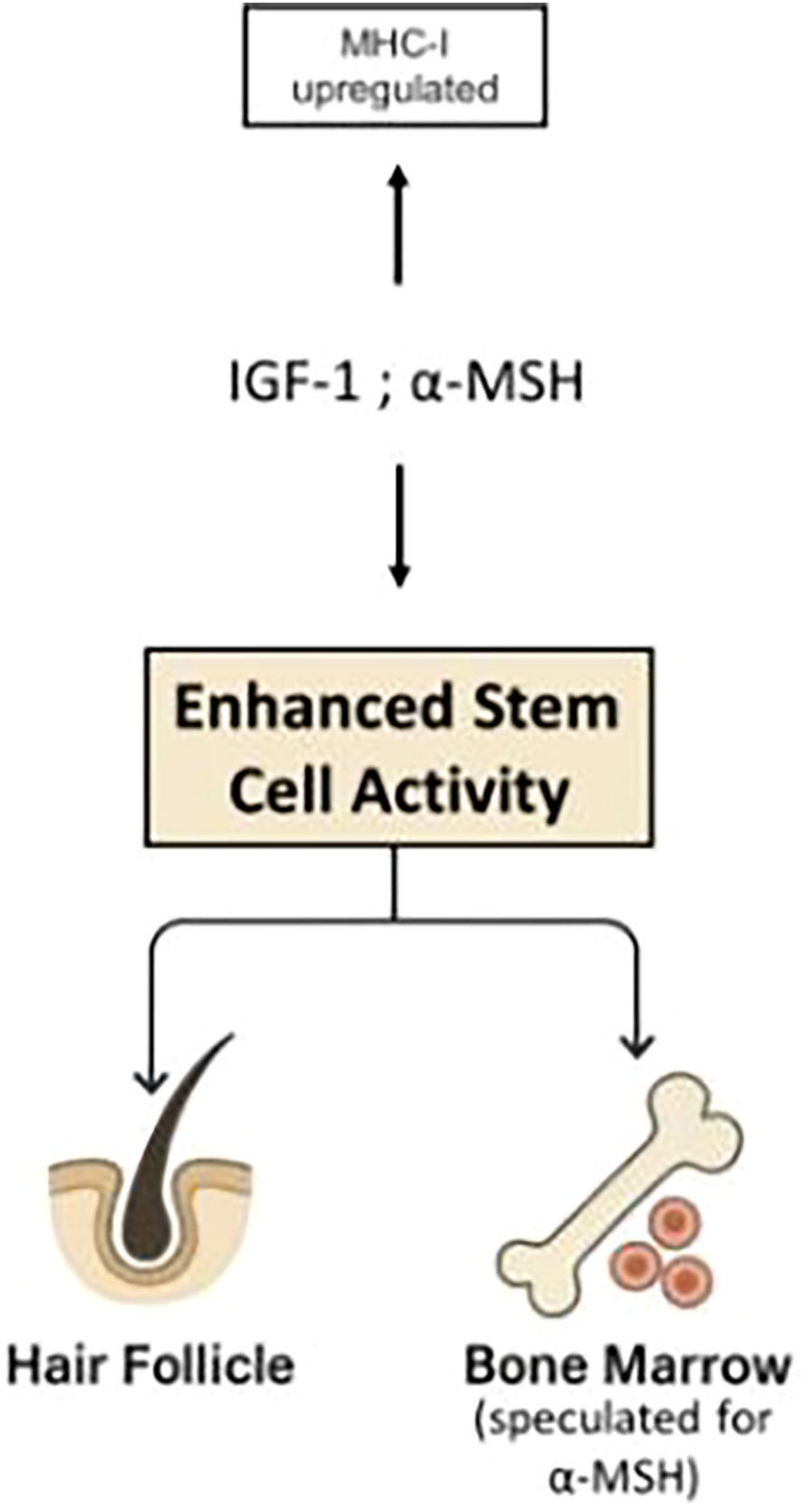
Agents such as α-MSH, IGF-1, which downregulate MHC-I expression in hair bulbs (9), also enhance stem cell renewal and proliferation (19–23). Both effects contribute to remission in alopecia areata.

The effects of these cytokines on the course of alopecia areata is a useful example. In normal anagen hair, *R* is constitutively positive which render *ΔM* positive and hair growth proceeds. When autoimmunity develops, the net sign of *ΔM* - positive or negative - will depend on the degree of stem cell activation, the intensity of the immune response, and the tissue’s susceptibility to immune attack - particularly the level of MHC-I expression. The relative contributions of each factor will determine whether symptoms manifest or whether the patient remains asymptomatic despite the presence of anti-follicular autoimmunity. Agents such as α-MSH, IGF-1, which downregulate MHC-I expression in hair bulbs (9), also enhance stem cell renewal and proliferation (19–23) (**Figure 5**). Both effects contribute to remission in alopecia areata. In contrast, IFN-γ upregulates MHC-I expression (9) thereby increasing the susceptibility to cytotoxic attacks and concurrently suppresses stem cell activity (13, 15–17), promoting relapses in alopecia areata. IFN-γ has been shown to induce the onset of AA in mice with activated immune cells (133). Although the preceding discussion omitted the effects of different cytokines on hematopoiesis for the sake of simplicity, these examples illustrate how various cytokines can influence relapses and remissions of AA. The modulation of stem cell activity by cytokines appears to play a pivotal role in triggering both remissions and relapses.

Internal or external factors that influence peripheral cytokine levels may trigger relapses or remissions. For example, viral infections linked to both the initial presentation and recurrence of AA include Epstein–Barr virus (EBV), hepatitis B virus (HBV), and hepatitis C virus (HCV) (139). Elevated plasma IFN-γ levels have been reported in EBV infection (140), acute HBV infection (141), and chronic HCV infection (142). Suppression of HFSC activity by high IFN-γ levels may contribute to the observed association between these viral infections and both the onset and relapse of AA. **Table 2** presents the effect of the four cytokines that modulate HF-IP on factors that potentially contribute to remission or relapse of AA.

**Table 2:** The effect of agents that modulate hair follicle immune privilege on the promotion of remissions (+) or relapses (-) in alopecia areata through their effects on MHC-I expression, HFSC activity, HSC activity and T and NK cell function.

	MHC-I expression	HFSC activity	HSC activity	T or NK cell function
IFN-γ	–	–	+	+/-
α-MSH	+	NA	–	+/-
IGF-1	+	+	–	+/-
TGF-β1 (high)	+	–	+	+/-
TGF-β1 (low)	+	–	–	+/-

IFN-γ, interferon γ; α-MSH, α-melanocyte stimulating hormone; IGF-1, insulin-like growth factor 1; TGF-β1, transforming growth factor β1.

According to **Table 2**, it is clear that the same agent can either promote remission or trigger a relapse in alopecia areata, depending on its effects on MHC-I expression, HFCS and HSC activities, and T and NK cell functions. Additionally, different cytokines and their varying concentrations can lead to similar or contrasting outcomes. This table highlights the role of stem cell activity in the remission or relapse of alopecia areata, illustrating the pleiotropic nature of these agents. It emphasizes how each agent’s impact on the disease is influenced by the presence and concentration of other agents.

Immune privilege collapse and restoration (6) may explain the onset and remission of symptoms in AIDs when the target organs are immune privileged. However, this mechanism does not account for fluctuations in disease severity when the affected organs lack immune protection, such as the intestinal barrier in IBD. The present article links stem cell status to flares and remissions in AIDs, regardless of whether the target organs are immune protected. For example, TGF-β1 has been shown to impair stem cell activity, cell proliferation, and secretory cell differentiation in Smad4-deficient intestinal organoids (143), while human IBD (and colon cancer) specimens show reduced Smad4 expression compared to healthy controls (144). These findings suggest that local TGF-β1 may suppress intestinal stem cell activity in IBD, thereby contributing to remission even in organs that are not normally immune privileged.

Taken together, the cell cycle phase of stem cells—strongly influenced by cytokines and growth factors in the tissue microenvironment—plays a central role in triggering flares in AIDs and in driving their resolution. However, as discussed earlier, an external supply of TGF-β will stabilize the quiescent phase and prevent such fluctuations, but remission is not expected. For example, although external TGF-β1 can restore IP in alopecia areata, hair regrowth will not occur due to TGF-β1–induced cell-cycle arrest, implying the absence of remission.

## Discussion

The present work introduces stem cell activity as a major contributor to the pleiotropic effects of IFN-γ and TGF-β1 and to the fluctuations of AIDs between flares and remissions. Integrating findings from multiple studies on hair follicle immune privilege, it proposes that immune privilege is not an inherent property of quiescent stem cells per se (104), but rather depends on the pathway driving quiescence. The TGF-β/Smad pathway not only induces quiescence in stem cells but also drives MHC-I downregulation, thereby explaining the association between stem cell quiescence and immune privilege. In contrast, IFN-γ induces quiescence while upregulating MHC-I expression. The effect of TGF-β on stem cells is long-lasting (102), though not irreversible. The present study suggests that the accumulation of Tregs in the hair bulge, as reported by Ali et al. (107), leads to high local concentrations of TGF-β1, which (a) induces quiescence in HFSCs within the bulge (31), and (b) downregulates MHC-I expression in these stem cells (9, 104), thereby conferring immune privilege to the bulge. It is important to note that quiescent stem cells do not undergo self-renewal while in the G_0_ phase. Therefore, according to Equation 1, they are highly susceptible to loss when exposed to immune attack (*R* < 0 when *Rdes* > 0), making protection from cytotoxic T cells essential for their survival.

At sites distant from the bulge, Treg frequency and TGF-β1 concentration decline to levels that promote HFSC proliferation and self-renewal (34), accompanied by increased MHC-I expression and heightened susceptibility of stem and epithelial cells to cytotoxic attacks, consistent with findings by Agudo (105). During the normal anagen phase, the TGF-β1 gradient supports hair growth by promoting stem cell differentiation outside the bulge while maintaining relative protection of the hair bulb. Notably, TGF-β1 levels low enough to enhance stem cell activity (pg/ml range) may still induce immune privilege. For example, in the gastric cancer tumor microenvironment, a TGF-β1 concentration of 21 pg/(mg protein) was associated with worse prognosis (145), likely due to TGF-β–mediated immune suppression (91) at these TGF-β1 levels. In alopecia areata, however, when immune privilege collapses, this same gradient renders cells outside the bulge susceptible to immune attack, contributing to follicular destruction. In contrast, the bulge stem cell reservoir is preserved owing to the high local concentration of TGF-β1. In normal follicles, externally supplied IFN-γ upregulates MHC-I expression in epithelial cells outside the bulge (9) and induces quiescence in HFSCs (13), thereby inhibiting hair elongation (17).

The proposed mechanism is supported by the report of no significant differences in the frequency of TGF-β+Tregs between alopecia areata patients and healthy controls, although the cell-to-cell contact and CD39 mediated activity of Tregs are impaired (109). Despite the lack of direct supporting evidence, the present work suggests that, as in normal hair, TGF-β1–secreting Treg cells remain abundant in the bulge during alopecia areata, thereby helping to preserve its immune privilege. Similar to hair bulge, HSC niches in the bone marrow are densely populated by Treg cells (110), which produce high levels of TGF-β1. As in the hair follicle, this creates a TGF-β1 concentration gradient between the niches and surrounding marrow (**Figure 1**), resulting in HSC quiescence within the niches and a relative activation outside them.

Stem cells outside the immune-protected sites such as hair bulge, or bone marrow HSC niches, may be affected by exposure to IFN-γ or TGF-β1. In inflammatory conditions and cancer, exposure to IFN-γ or to high concentrations of TGF-β1 restrain the immune attack by downregulating HSC activity in bone marrow (15, 16, 34) and by slowing down hematopoietic progenitor cell differentiation in lymph nodes (146), while α-MSH and IGF-1 promote the immune attack by accelerating HSC renewal and proliferation (19, 20). Different combinations of these cytokines, as well as different concentrations will either promote or suppress stem cell activity thus contributing to the pleiotropic effect of IFN-γ and TGF-β1 in AIDs, infections and cancer, and to the pleiotropic effects of α-MSH and IGF-1 on T cell or NK cell function (**Table 1**). However, high concentrations of TGF-β1 secreting Tregs in sites like hair follicle bulge or HSC niches that persist in inflammations (and probably in advanced cancer) contribute to the preservation of dormancy and stemness of these cells even when exposed to agent like IFN-γ.

In AIDs, the complex interplay between stem cell activity and cytokines in both target tissues and the bone marrow—governing the production of tissue and immune cells—plays a key role in driving relapses and remissions.

Despite the complex mix of positive and negative effects exerted by the agents listed in **Table 1** on AID progression, their impact on stem cell activity emerges as most prominent. **Table 3** is derived from **Table 1** and summarizes the effects of the five agents on *Rreg*, *Rdes*, and *R* values, as well as on the clinical course of AIDs. The *Rreg* value reflects stem cell activity in the target tissue. Since the effects of each agent on HFSC, MSC, and HSC activity, whether stimulatory or inhibitory, are comparable (**Table 1**), it is assumed that similar effects occur in stem cells of other target tissues. The *Rdes* value represents the intensity of the autoimmune attack. Accordingly, the effect of each agent on *Rdes* is inferred from its impact on immune cell activity, as shown in **Table 1** (i.e., whether it upregulates or downregulates immune responses). **Table 3** reveals a clear correlation between clinical outcome—beneficial or pathogenic—and the impact of these agents on *Rreg*. *An increase in Rreg value is associated with symptom improvement, whereas a decrease in Rreg value corresponds to worsening disease. This correlation underscores the central role of stem cell activity in AID pathogenesis.* The correlation is specific to AIDs and not observed in other inflammatory conditions, since the direct autoimmune attack on target tissues that characterizes AIDs necessitates robust stem cell activity to replace damaged tissue and alleviate symptoms. Moreover, this correlation suggests that the inflammatory response associated with autoimmunity has a relatively limited effect on *Rreg*.

**Table 3:** Effects of various agents on Rreg, Rdes, R, and AID outcomes.

Agent	Effect on *Rreg value*	Effect on *Rdes value*	Effect on *R* value (Equation 1)	Effect on AIDs
IFN-γ	decrease	decrease/increase	decrease/increase	pathogenic (animal models)
α-MSH	increase	increase/decrease	increase/decrease	beneficial
IGF-1	increase	increase	increase/decrease	beneficial
Tacrolimus	increase (at high levels)	decrease	increase	beneficial
TGF-β1	increase/decrease	increase/decrease	increase/decrease	beneficial/pathogenic

IFN-γ, interferon γ; α-MSH, α-melanocyte stimulating hormone; IGF-1, insulin-like growth factor 1; TGF-β1, transforming growth factor β1.

Although an association between clinical outcome and impact on *Rreg* value is clearly presented by the data, causality is not evident. Three possible mechanisms may account for the observed association: (a) the agent-induced change in *Rreg* directly drives the effect on symptoms; (b) changes in disease activity secondarily alter *Rreg;* or (c) the agent independently affects both *Rreg* and disease symptoms. An experiment that may help in assessing causality can use an animal model where the autoimmune disease is induced by impairing Tregs function. For example, neutralization of circulating IL-2 with an anti–IL-2 monoclonal antibody for a limited period induces autoimmune gastritis in BALB/c mice. In diabetes-prone nonobese diabetic (NOD) mice, similar treatment accelerates the onset of diabetes and triggers a broad spectrum of T cell–mediated autoimmune diseases, including gastritis, thyroiditis, and sialadenitis (147). It would be instructive to examine whether stem cell activity in the target tissues of these mice is reduced following monoclonal antibody treatment. If no reduction is observed, mechanism (b) can be excluded. Targeting stem cells directly may help determine whether mechanism (a) accounts for the association between *Rreg* and AID symptoms. This could be tested by treating healthy or disease-prone NOD mice with anti-LGR5 antibodies (148, 149) and assessing whether inflammatory diseases develop. The induction of inflammation under these conditions would support mechanism (a). Conversely, if neither (a) nor (b) is implicated, mechanism (c) would be the most likely driver of the observed association.

Equations 1 and 3 provide a basic framework linking the occurrence of flares and remissions in AIDs to the rates of target tissue regeneration and destruction. Their validity was supported by two earlier studies. One demonstrated that the refractoriness of four autoimmune diseases to steroids and immunosuppressive drugs results from near-complete loss of secreting cells combined with the extremely low regenerative capacity of the affected tissues (152). Another showed that a high recovery rate of target tissue accounts for the remitting–relapsing disease pattern, the presence of autoantibodies in healthy individuals, and the responsiveness to immunosuppressive therapy. Furthermore, analyzing individual AIDs through the balance between tissue destruction and regeneration yielded key insights—for example, explaining the difference between androgenic alopecia, a non-remitting disease, and alopecia areata, a remitting–relapsing AID (1). Collectively, these earlier findings support the validity of the equations used in the present study.

## Summary

The present work introduces stem cell activity as a major contributor to the pleiotropic effects of IFN-γ and TGF-β1, as well as to the fluctuations of autoimmune diseases between flares and remissions. Drawing on findings from studies of hair follicle immune privilege, it is proposed that immune privilege is not an intrinsic property of quiescent stem cells, as previously suggested, but rather depends on the signaling pathways driving quiescence. Although both IFN-γ and high levels of TGF-β1 induce stem cell quiescence, they exert opposite effects on immune privilege: IFN-γ upregulates MHC-I expression, whereas TGF-β1 downregulates it. This principle applies to both immune-protected sites—target organs such as hair follicles and hematopoietic stem cell niches in the bone marrow (**Figure 2**). In addition, cytokines such as IGF-1 and α-MSH, which enhance stem cell activity, also downregulate MHC-I expression (**Figure 5**). Different combinations and concentrations of these four cytokines can either stimulate or suppress stem cell activity in bone marrow and target organs, and either preserve or disrupt immune privilege. This interplay underlies the pleiotropic nature of all four cytokines.

Two mechanisms are proposed to account for the pleiotropic effects of IFN-γ and TGF-β1. The first involves their opposing influences on the activity of bone marrow–resident immune cells, with IFN-γ and high levels of TGF-β1 exerting contrasting effects. The second relates to the differential impact of IFN-γ on the intensity of immune attack in the bone marrow versus the target tissue during autoimmune disease. Stem cell activity within target organs is also linked to the clinical dynamics of AIDs: high stem cell activity promotes tissue regeneration following autoimmune attack and contributes to remission, whereas stem cell quiescence, together with immune-mediated tissue destruction, drives disease flares. A clear correlation emerges between the influence of various agents on stem cell activity and clinical outcomes in AIDs, underscoring the central role of stem cell activity in AID pathogenesis. This work further proposes that a TGF-β1 gradient—between the hair follicle bulge and the regions outside it, or between HSC niches at the bone endosteal surface and surrounding marrow (**Figure 1**) —allows simultaneous immune protection of the stem cell reservoir and active regeneration in less protected areas. During autoimmune disease, this reservoir remains shielded and may provide new cells to repair damaged tissue.

## Data Availability

The original contributions presented in the study are included in the article/supplementary material. Further inquiries can be directed to the corresponding author.
